# Are Community-Level Financial Data Adequate to Assess Population Health Investments?

**DOI:** 10.5888/pcd9.120066

**Published:** 2012-08-09

**Authors:** Tim Casper, David A. Kindig

**Affiliations:** Author Affiliation: Tim Casper, Madison College, Madison, Wisconsin.

## Abstract

The variation in health outcomes among communities results largely from different levels of financial and nonfinancial policy investments over time; these natural experiments should offer investment and policy guidance for a business model on population health. However, little such guidance exists. We examined the availability of data in a sample of Wisconsin counties for expenditures in selected categories of health care, public health, human services, income support, job development, and education. We found, as predicted by the National Committee on Vital and Health Statistics in 2002, that availability is often limited by the challenges of difficulty in locating useable data, a lack of resources among public agencies to upgrade information technology systems for making data more usable and accessible to the public, and a lack of enterprise-wide coordination and geographic detail in data collection efforts. These challenges must be overcome to provide policy-relevant information for optimal population health resource allocation.

## Background

Because of the increasing attention on improving population health, policy makers in the public and private sectors are asking for better information to guide their investment decisions. The Institute of Medicine (1) has called on the federal government to issue an annual report on trends and disparities in social and environmental factors that affect health and to advance the use of system-based simulation models to explain the health consequences of the underlying determinants of health. These requests are consistent with calls for comparative effectiveness research to inform cost-effective allocation of resources among the multiple determinants of health (2,3).

Much of the variation in health outcomes among communities may result from different levels of financial and nonfinancial policy investments over time; these natural experiments should offer investment guidance for a business model of population health (4). Why have researchers not estimated the per capita level of investment at the community level for each of the determinants of health (medical care, public health, behaviors, and the social and physical environment and their related programs and policies) beyond which health does not improve at all or very much? Why haven’t researchers suggested benchmarks for each community according to its level of health and health-producing factors?

Almost nothing in the public and population health literature addresses these questions. Some empiric research has attempted to determine the balance of health determinants associated with various health outcomes; such work examines the factor itself — such as being uninsured or high school graduation rates (5) — but not the financial or policy resources producing them. One exception is the work of the Trust for America’s Health: it estimated that an investment of $10 per person per year in proven community-based programs to increase physical activity, improve nutrition, and prevent smoking and other tobacco use could save more than $16 billion annually within 5 years (6).

Other research explores health investment relationships in detail, estimating the effect of public services on state mortality while controlling for median income and income inequality (7). Total per capita expenditures in public services were significantly associated with all mortality measures, as were expenditures in education, environment, and housing. Similarly, a study of the effects of state expenditures on state-level age-adjusted mortality reported on public expenditures, tax structures, and welfare program rules and found that more generous education spending, more progressive tax systems, and more lenient welfare program rules helped to improve population health (8). However, the magnitudes of the effects were small, most likely because using the state as the unit of analysis masks much of the variation in outcomes and investments at local levels.

In *Shaping a Health Statistics Vision for the 21st Century*, the National Committee on Vital and Health Statistics (9) called for a better health statistics information system to help policy makers decide how to use health resources and emphasized the importance of considering factors beyond the health of the individual, to include community and socioeconomic factors. We do not believe, however, that there yet exists a national or state data set that includes comparable measures of financial investment in the multiple determinants of population health at the substate level. The absence of such a data set is probably due to the many challenges of collecting data from multiple levels of government and making them comparable.

As part of its annual reports on state expenditures, the National Association of State Budget Officers noted that comparing data, even among states, is difficult because data are collected and reported differently (10). For example, they noted that spending on juvenile corrections may be characterized as spending on corrections in 1 state and spending on human services in another.

## Exploring the Challenge of Comparable Data at the County Level

To explore the challenge of comparable data, we examined expenditure data in 10 counties in Wisconsin in the spring of 2011. The selected urban, suburban, and rural counties represented nearly 40% of Wisconsin’s population. We searched public websites and contacted staff at state, county, and private agencies to inquire about the availability of data on total expenditures (federal, state, local, and private) and the number of people served. We evaluated the data according to whether they reflected all possible expenditures for a particular program and all possible people served. Our objective was to find comparable data at the county level in 6 categories: health care, public health, human services, income support, job development, and education.

### Health care

The Wisconsin Department of Health Services publishes regular reports on enrollment in Medicaid programs offered in Wisconsin (www.dhs.wisconsin.gov/aboutdhs/opib/policyresearch/ClientsServed.pdf ). These reports, which do not appear to be continuously updated, identify the number of people served in each program statewide. The department provided information on the number of people in each program by county, including the Community Health Improvement Process program and Medicaid long-term care waivers, for 1 calendar year. However, people enrolled through the statewide enrollment center were not identified by county of residence. Additionally, 7 of the state’s 11 Native American tribes enroll qualifying participants into Medicaid programs but do not identify enrollees by county of residence; instead, such participants are identified by tribe.

### Public health

Local health departments in Wisconsin are organized at the county and municipal level. All local health departments are required to report to the state on their annual expenditures from all sources of revenues, including gifts, in 5 basic service areas: communicable disease surveillance, prevention, and control; generalized public health nursing program; health promotion; chronic disease prevention; and human health hazard prevention and control. Local health departments do not report per capita expenditures by service area. Instead, the per capita expenditures are calculated according to total expenditures for all service area activities. Calculations are made in this way because such expenditures promote the health of the entire county or municipality, not just individuals. Also, because local health departments are identified according to 3 levels of service, with each level offering a different set of services (level I provides the most basic and level III the most advanced), data on per capita expenditures are not comparable across all counties.

### Human services

Wisconsin has 2 reporting systems for data on expenditures in human service programs for people who have developmental disabilities, mental health issues, alcohol or other drug abuse issues, or physical or sensory disabilities. One is the Human Services Reporting System, which organizes expenditure data by standard program categories (eg, respite care, daily living skills training). This system, which permits the calculation of per capita expenditures by disability or issue group in each county, is not available electronically because it is based on COBOL (Common Business-Oriented Language) and produces only paper reports. The other system is the Human Services Revenue Report, which organizes expenditures data by disability or health issue group and revenue source. Its purpose is to allow counties to report exact costs to the state. It does not include data on the number of people served according to disability or issue group, so it does not permit calculation of per capita expenditures. Neither report includes data on all expenditures in human services, nor does either report include data on services provided by Medicaid health maintenance organizations or private providers through Medicaid fee-for-service.

These reports have shortcomings. The sum of expenditures identified in the 2 reports should be identical; however, Wisconsin Department of Health Services staff reported that the information is not always identical because different staff people may be responsible for completing the 2 reports. Additionally, some counties jointly administer human service programs through a single service agency and jointly report data on enrollment and expenditures, so these data are not reported by county but by agency. Finally, neither report includes data on human service expenditures made through FamilyCare, a program that delivers long-term care to the elderly and people who have disabilities. FamilyCare is delivered by consortia of counties or by nonprofit managed-care organizations (MCOs) that serve multiple counties. The publicly available data on FamilyCare enrollment identify the number of people who receive services from the consortium or MCO by county, but the data do not identify people who receive services but reside outside the service region (nonresidents), which is permitted by law. These nonresident participants are not identified by their county of residence. The data also do not identify the level of capitated payment received by the consortium or MCO that serves the nonresident. That is, does the consortium or MCO serving a nonresident receive a level of payment equal to the nonresident’s county of residence or is it same level of payment that the consortium or MCO receives for serving its county’s residents?

### Income supports

#### Earned income credit

Wisconsin administers an earned income tax credit that functions similarly to the federal earned income tax credit except that people who have no children are not eligible. The Wisconsin Department of Revenue generates annual reports on the number of people who receive the Wisconsin credit and the per capita credit amount for each county, but it does not publish information on the number of people who receive the federal earned income tax credit.

#### Special Supplemental Nutrition Program for Women, Infants, and Children

Wisconsin maintains enrollment data on the Special Supplemental Nutrition Program for Women, Infants, and Children (WIC) program by county. At one time, the state requested data from local WIC providers on private support used to operate the WIC program, but it no longer does so because the data were reported inconsistently. Currently, the WIC program does not publish regular public reports on the number of people served by WIC or WIC expenditures by county.

#### FoodShare

FoodShare Wisconsin, formerly known as Food Stamps, maintains data on the number of people served by county. However, participants who enroll through a statewide center are not identified by county of residence, nor do counties know how many, if any, of these recipients reside in their county. The state is interested in enrolling more participants through the statewide center because it is seen as more efficient. Participants can enroll online through the statewide center or visit their county human services agency for enrollment assistance. Because FoodShare is a federally funded program, the state is required to maintain data on total expenditures in program benefits.

### Job development

We did not find any data set that tracked local job development activity.

### Education

We identified 3 data sets on expenditures for education for kindergarten through grade 12.

#### National Center for Education Statistics

The National Center for Education Statistics annually publishes statistics on expenditures by school district and revenue source — federal, state, local, and program. It also calculates the per-student expenditure for each district. These data present 2 challenges. First, Wisconsin school districts may be organized in 1 of 3 ways: kindergarten through grade 12, kindergarten through grade 8, and Union High School districts, which serve only grades 9 through 12. Each district type has its own cost structure. Second, Wisconsin has 426 school districts. Many districts, particularly in rural parts of the state, cross county boundaries.

#### Child nutrition expenditures

School districts report the amount that they spend on student meals to the Wisconsin Department of Public Instruction. The school districts also report revenues from federal and state subsidies and program revenues associated with meal payments from students and nonstudents and meals prepared by the district for a third party, such as another school district, through contracts. The per-student meal expenditure is based on revenues from all such services. Expenditure data exist for all public school districts and for private school districts that participate in the federal and state subsidy programs. These data are not available electronically because the COBOL-based system that hosts the data produces only paper reports.

#### Head Start and Early Head Start

The US Department of Health and Human Services (HHS) maintains information on federal expenditures for Head Start and Early Head Start and the number of children served by the programs. Wisconsin provides supplemental Head Start grants that providers may use as matching funds required by the federal program. Wisconsin maintains data only on the number of children served through its own grants. It does not maintain data on how much of each supplemental grant is used as federal matching funds, nor does it or HSS request information on other private or public resources used to support these programs.

## Conclusion

The National Committee on Vital and Health Statistics identified challenges related to the collection of data on health statistics. The challenges include difficulty in locating useable data, a lack of resources among public agencies for upgrading information technology systems to support greater public access to data, and a lack of enterprise-wide coordination and geographic detail in data collection efforts. To this could be added the political barriers for developing common standards among state and community jurisdictions.

Wisconsin data on population health expenditures are often limited by 1 or more of these challenges. If researchers, administrators, and the public are to analyze and evaluate programs and influence policy makers, much work is needed to make data accessible and comparable. Expenditure data must be collected for all programs and their funding sources, and accurate counts must be made of the people served by such programs by geographic unit. Upgrading information technology systems to provide data in a format that is easy to use and manipulate will require investments of money and staff resources. Data collection efforts need to be integrated horizontally (among state agencies, for example) and vertically (among federal, state, and local funding sources, for example).

We live in a resource-limited world that has disparities in health outcomes by race, sex, socioeconomics, and geography. Public and private policy makers at the national, state, and local level want to find resources to address these disparities. But as Evans and Stoddart say, “Redirecting resources means redirecting [someone’s] income[s]. . . . [M]ost students of population health cannot confidently answer the question ‘Well, where would *you* put the money?’” (11). Standardizing data is not the most exciting activity, but making the most cost-effective investments requires it (4). It is time for state and national government to make progress toward this objective, perhaps under the leadership of the new HSS Community Health Data Initiative (12). Improving our health cost effectively may depend on it.
